# Cellulase production and efficient saccharification of biomass by a new mutant *Trichoderma afroharzianum *MEA-12

**DOI:** 10.1186/s13068-021-02072-z

**Published:** 2021-11-22

**Authors:** Zhi-Qing Peng, Chuang Li, Yi Lin, Sheng-Shan Wu, Li-Hui Gan, Jian Liu, Shu-Liang Yang, Xian-Hai Zeng, Lu Lin

**Affiliations:** 1 College of Energy, Xiamen University, Xiamen, 361102 China; 2 Fujian Engineering and Research Centre of Clean and High-Valued Technologies for Biomass, Xiamen, 361102 China; 3 Xiamen Key Laboratory of Clean and High-Valued Utilization for Biomass, Xiamen, 361102 China

**Keywords:** *Trichoderma afroharzianum*, Compound mutagenesis, Adaptive laboratory evolution, Cellulase, Biomass

## Abstract

**Background:**

Cellulase plays a key role in converting cellulosic biomass into fermentable sugar to produce chemicals and fuels, which is generally produced by filamentous fungi. However, most of the filamentous fungi obtained by natural breeding have low secretory capacity in cellulase production, which are far from meeting the requirements of industrial production. Random mutagenesis combined with adaptive laboratory evolution (ALE) strategy is an effective method to increase the production of fungal enzymes.

**Results:**

This study obtained a mutant of *Trichoderma afroharzianum* by exposures to N-methyl-N’-nitro-N-nitrosoguanidine (MNNG), Ethyl Methanesulfonate (EMS), Atmospheric and Room Temperature Plasma (ARTP) and ALE with high sugar stress. The *T. afroharzianum* mutant MEA-12 produced 0.60, 5.47, 0.31 and 2.17 IU/mL FPase, CMCase, pNPCase and pNPGase, respectively. These levels were 4.33, 6.37, 4.92 and 4.15 times higher than those of the parental strain, respectively. Also, it was found that *T. afroharzianum* had the same carbon catabolite repression (CCR) effect as other *Trichoderma* in liquid submerged fermentation. In contrast, the mutant MEA-12 can tolerate the inhibition of glucose (up to 20 mM) without affecting enzyme production under inducing conditions. Interestingly, crude enzyme from MEA-12 showed high enzymatic hydrolysis efficiency against three different biomasses (cornstalk, bamboo and reed), when combined with cellulase from* T. reesei* Rut-C30. In addition, the factors that improved cellulase production by MEA-12 were clarified.

**Conclusions:**

Overall, compound mutagenesis combined with ALE effectively increased the production of fungal cellulase. A super-producing mutant MEA-12 was obtained, and its cellulase could hydrolyze common biomasses efficiently, in combination with enzymes derived from model strain* T. reesei*, which provides a new choice for processing of bioresources in the future.

**Supplementary Information:**

The online version contains supplementary material available at 10.1186/s13068-021-02072-z.

## Background

In biorefinery process, cellulase plays a key role in converting cellulosic biomass into fermentable sugar to produce chemicals and biofuels [1]. Many microorganisms are known to produce cellulase including bacteria, fungi and actinomycetes [2–4]. Among microbial sources, filamentous fungi have higher secretory capacity and considered to be the most promising cellulase producers [3]. However, most of the filamentous fungi obtained by natural breeding have incomplete enzyme systems, which are far from meeting the requirements of industrial production [5], so the selection of strains with high yield and quality of cellulase is essential to expand the application range of them.

Classic physical or chemical mutagenesis is a kind of strategy that can effectively improve the performance of strains, especially for increasing enzyme yields [6, 7]. The usual reported mutagenesis techniques are UV radiation, Heavy ion, Atmospheric and Room Temperature Plasma (ARTP), N-methyl-N’-nitro-N-nitrosoguanidine (MNNG) and Ethyl Methanesulfonate (EMS) [8–12]. Chemical mutagenesis can cause mutations in microorganisms by adding chemical mutagens, which is highly specific to DNA [13]. ARTP is a recently developed whole-cell mutagenesis tool based on radio frequency atmospheric pressure glow discharge plasma. The active particles produced by ARTP act on microorganisms to change the permeability of cell membranes, then, active particles and oxidative free radicals enter the cells and interact with biological macromolecules such as DNA and proteins to cause direct and/or indirect damage to DNA [14]. The use of single mutagenesis or simple compound mutagenesis treatment has been reported [11, 12], whereas MNNG/EMS/ARTP has not.

Adaptive laboratory evolution (ALE) also can achieve the phenotypic optimization. ALE refers to the realization of microbial evolution by imposing human interference and controlling the growth environment of microbes [15]. Compared with metabolic engineering, ALE does not need to consider the intricate and intersecting metabolic network of microorganisms [16]. It only needs to design the corresponding interference factors according to the target. In this case, most of the cellulase-producing *Trichoderma* spp. have catabolite repression [17]. High concentration of metabolic repressors produced during the fermentation process greatly hinders the production of cellulase [17]. Therefore, it is possible to combine mutagenesis with ALE and artificially adjust the screening medium components and the concentration of metabolic repressors contained at different mutagenesis stages to improve the enzyme production.

In this study, MNNG/EMS/ARTP mutagenesis combined with ALE method was used to improve cellulase production in *T. afroharzianum*. Subsequently, it was found that the mutant can tolerate the inhibition of glucose (up to 20 mM) without affecting enzyme production under inducing conditions. Interestingly, crude enzyme from the mutant showed high enzymatic hydrolysis efficiency against three common biomasses (cornstalk, bamboo and reed), which were pretreated by cooking with active oxygen and solid alkali (CAOSA), when combined with cellulase from *T. reesei* Rut-C30. In addition, real-time reverse transcription quantitative PCR (RT-qPCR) was employed to analyze the transcription levels of major cellulase genes and transcription factors genes in isolated mutant.

## Results and discussion

### Development of a compound mutagenesis combined with ALE method

**Fig. 1:**
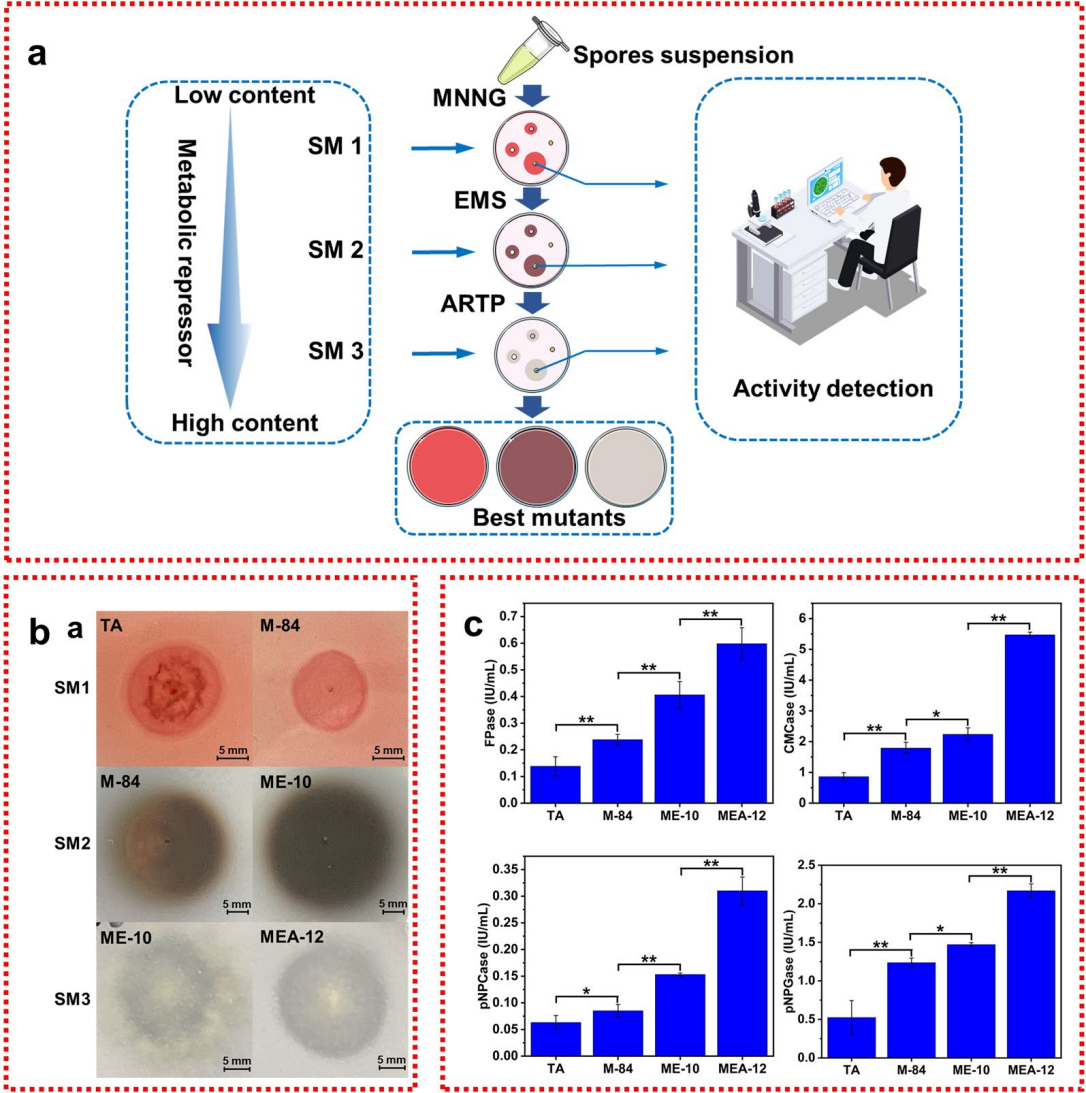
Scheme of mutation procedure (**a**), phenotypic investigation of *T. afroharzianum* and its mutants, grown on selection medium **b** and their cellulase activities in fermentation medium (**c**). TA is a wild type of *T. afroharzianum*; M-84 is the best mutant in first round of mutagenesis; ME-10 is the best mutant in second round of mutagenesis; MEA-12 is the best mutant in third round of mutagenesis. Error bars indicate the mean ± SD of three biological replicates and statistical significance was calculated by Student’s *t* test (* (*p* < 0.05) and ** (*p* < 0.01))

To optimize the screening efficiency of high-yield cellulase mutants, a strategy of compound mutagenesis combined with microbial adaptive laboratory evolution (ALE) was established (Fig. 1a). By artificially adjusting the components of screening medium (SM 1–3) and the concentration of environmental factors (such as metabolic repressors) in different mutagenesis stages, the ability of filamentous fungi to produce enzymes is improved. Compared with previous mutagenesis methods, the new strategy provides not only different mutagenesis sources, but also a screening medium corresponding to each round of mutagenesis, which has strong purpose and high practicability. As the mutagenesis round progressed, the proportion of mutants with high cellulase activity in the total mutants increased (Table 1). Specifically, in the first round of mutagenesis, a modified classic cellulose-congo red medium was used for preliminary screening [18]. Both the wild-type and the mutant M-84 colonies showed clear zones around colonies, and the clear zones around the mutant M-84 colonies became slightly larger (Fig. 1b). In the second round of mutagenesis, the modified esculin medium was used for preliminary screening [19]. The glycosidic bond of esculin was destroyed by β-glucosidase and complexed with ferric citrate to form a brown-black substance [19]. The brown-black substance formed by ME-10 colonies was darker and larger than that of mutant M-84 (Fig. 1b). In the third round of mutagenesis, the modified MCC medium was used for preliminary screening. The clear zones around colonies of mutant MEA-12 were larger than that of ME-10 (Fig. 1b). Inoculated in the fermentation medium, the cellulase activity of the above apparent positive mutant strains was significantly improved (Fig. 1c). These data confirmed that the compound mutagenesis combined with microbial ALE strategy was an effective method for screening high-producing cellulase strains.

**Table 1:** Number and positive mutation rate of wild strain *T. afroharzianum* obtained by three rounds of mutagenesis

Mutagenic round	Mutagenic method	Total number of mutants	Number of strains screened by enzyme activity				Positive mutation rate (%)
			−	+	++	+++	
Round 1	MNNG	84	60	19	4	1	28.57
Round 2	EMS	102	75	17	6	4	26.47
Round 3	ARTP	86	61	11	8	6	29.07

“−” refers to the negative mutants; “+” refers to the mutants with low activity; “++” refers to the mutants with middle activity; “+++” refers to the mutants with high activity

### Mutagenesis and screening

**Fig. 2:**
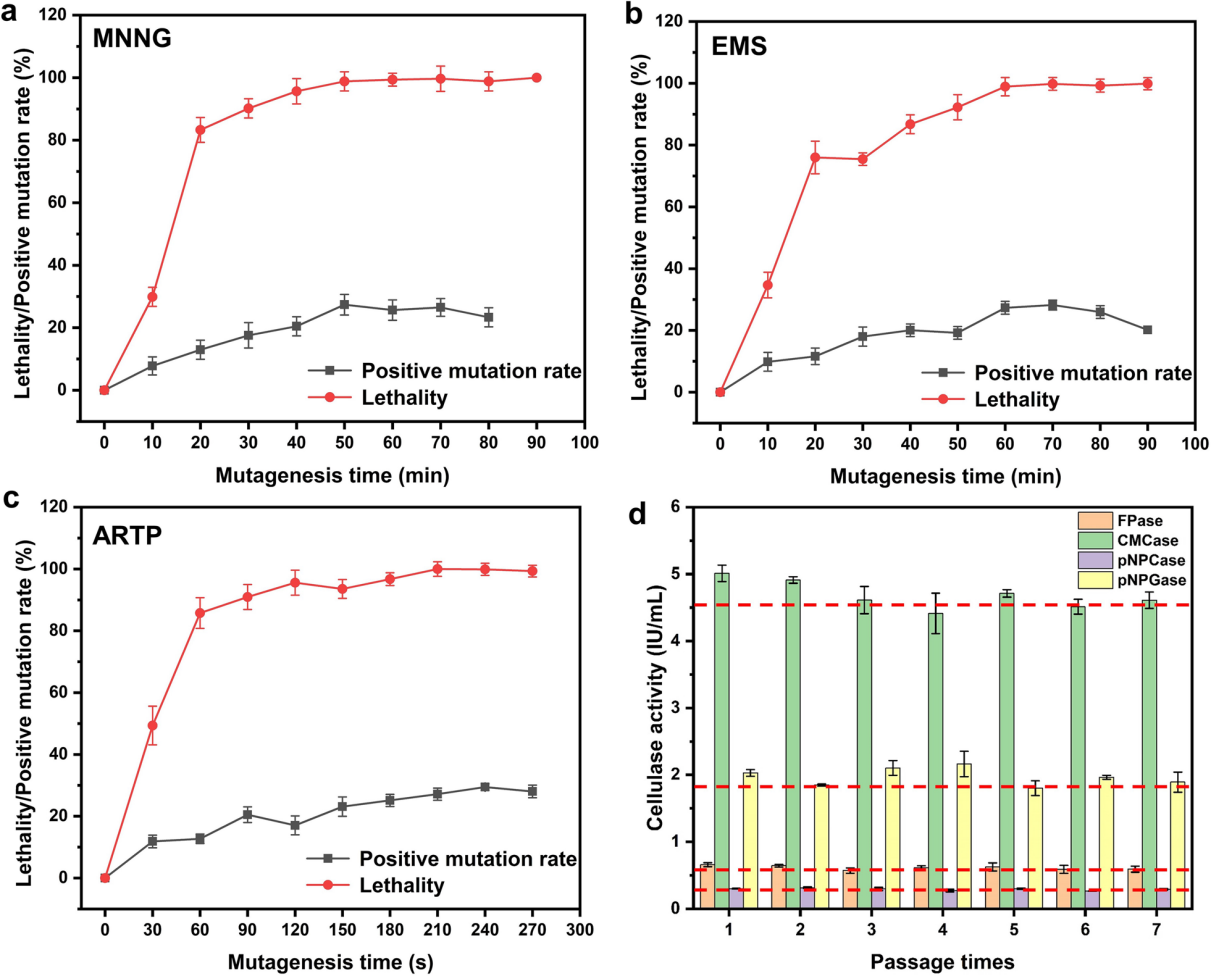
Lethality and positive mutation rate of *T. afroharzianum* in each round of mutagenesis **a**–**c** and subculture stability of high-yielding mutant MEA-12 under the inducing condition (**d**). Error bars indicate the mean ± SD of three biological replicates

The mutagenesis was performed in three rounds: (1) one round of MNNG, (2) one round of EMS, and (3) one round of ARTP. Before each round of mutagenesis, a preliminary experiment on the relationship between lethality and positive mutation rate was done. In each round of mutagenesis, with the increased of time, the lethality rate of the strain increased, while the positive mutation rate reached a peak. In the first round of MNNG mutagenesis, when the mutagenesis time was 50 min, the lethality rate of the strain exceeded 99% (Fig. 2a). The lethality rate was resulted in 100% when the mutagenesis time reached 90 min (Fig. 2a). Combining with the positive mutation rate curve, it indicated that the optimal treatment time for the first round of mutagenesis was 50 min. Similarly, the optimal treatment time for the second round of EMS mutagenesis was 70 min and the optimal treatment time for the third round of ARTP mutagenesis was 240 s (Fig. 2b, c).

In the first round of MNNG mutagenesis, a total of 84 mutants were obtained (Table 1). Combined with the SM1 for preliminary screening, there were 24 colonies with a higher diameter ratio between each colony and its clear zone than those of the wild type. Among them, 12 mutants’ cellulase activity in the re-screening were higher than the wild type. The mutant with the highest enzyme activity was numbered 84, named M-84 (Additional file 1: Fig. S1). After the second round of EMS mutagenesis, a total of 102 mutants were obtained (Table 1). Combined with the SM2 for preliminary screening, there were 27 colonies with darker color and larger diameter than those of the M-84. Among them, 4 mutants’ cellulase activity in the re-screening were higher than those of the M-84. The mutant with the highest enzyme activity was numbered 10, named ME-10 (Additional file 1: Fig. S2). After the third round of ARTP mutagenesis, the total number of mutants obtained was 86 (Table 1). Combined with the SM3 for preliminary screening, there were 25 colonies with a higher diameter ratio between each colony and its clear zone than those of the ME-10. Among them, the cellulase activities of 18 mutants in the re-screening were higher than those of the ME-10. The mutant with the highest enzyme activity was numbered 12, named MEA-12 (Additional file 1: Fig. S3).

After multiple rounds of mutagenesis, the cellulase of MEA-12 was significantly improved compared with the wild type (FPase increased 4.33-fold, CMCase increased 6.37-fold, pNPCase increased 4.92-fold, pNPGase increased 4.15-fold). This appears to be attributable to the compound mutagenesis combined with microbial ALE strategy, which avoided the defect that the repeated processing of a single mutagenesis technique leading to reducing the efficiency of mutagenesis [20]. The selected substrates (CMC-Na, esculin, MCC) at each stage can improve the cellulase activity of the target strain.

To evaluate the genetic stability of the mutant MEA-12, the cellulase activity was measured in the fermentation medium after seven consecutive subcultures. No significant change in cellulase activities was observed after the subcultures (Fig. 2d).

### Cellulase activity analysis of the mutant MEA-12

**Fig. 3:**
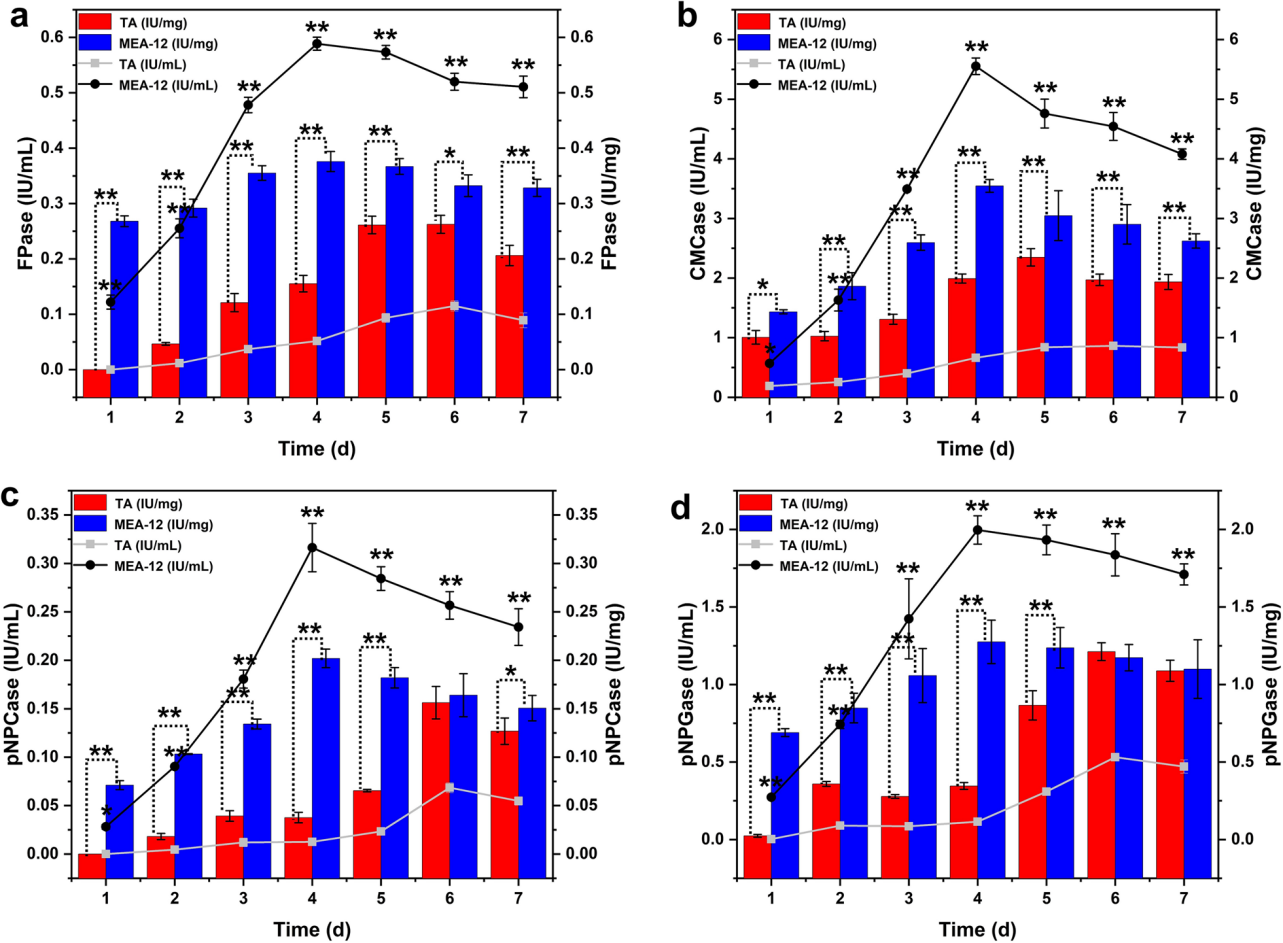
Activities of FPase, CMCase, pNPCase and pNPGase from TA and MEA-12. “IU/mL” means the specific activities which are calculated based on volume while the “IU/mg” is based on protein mass. Error bars indicate the mean ± SD of three biological replicates and statistical significance was calculated by Student’s *t* test (* (*p* < 0.05) and ** (*p* < 0.01))

Cellulose degradation is attributed to the synergistic action of three complementary enzyme activities: (1) endoglucanases (EGs); (2) cellobiohydrolases (CBHs); (3) β-glucosidases (BGs) [21]. In this study, a cellulase high-producer MEA-12 was obtained by compound mutagenesis combined with microbial ALE method. To further compare the difference in the enzyme production between parental strain and mutant strain, the fermentation time for optimal cellulase production was measured. The results showed that the enzyme activity of the parental strain *T. afroharzianum *(TA) reached the maximum on the day 6, while that of the mutant MEA-12 was on the day 4 (Fig. 3). This will greatly shorten the fermentation cycle of enzyme production and reduce production costs.

Moreover, the specific activities (IU/mg) of strain MEA-12 which were calculated based on protein mass increased 1.44, 1.51, 1.29 and 1.05 times in FPase, CMCase, pNPCase and pNPGase, respectively, compared with that of the parental strain (Fig. 3). The increase of enzyme activity and enzyme protein per unit volume can enhance the comprehensive activity of enzymes [22], which means that the increased cellulase activities in mutant MEA-12 is due to both the improved enzyme activity and enzyme production, and based on data, the former was higher than the latter.

### Glucose tolerance of the mutant MEA-12

**Fig. 4:**
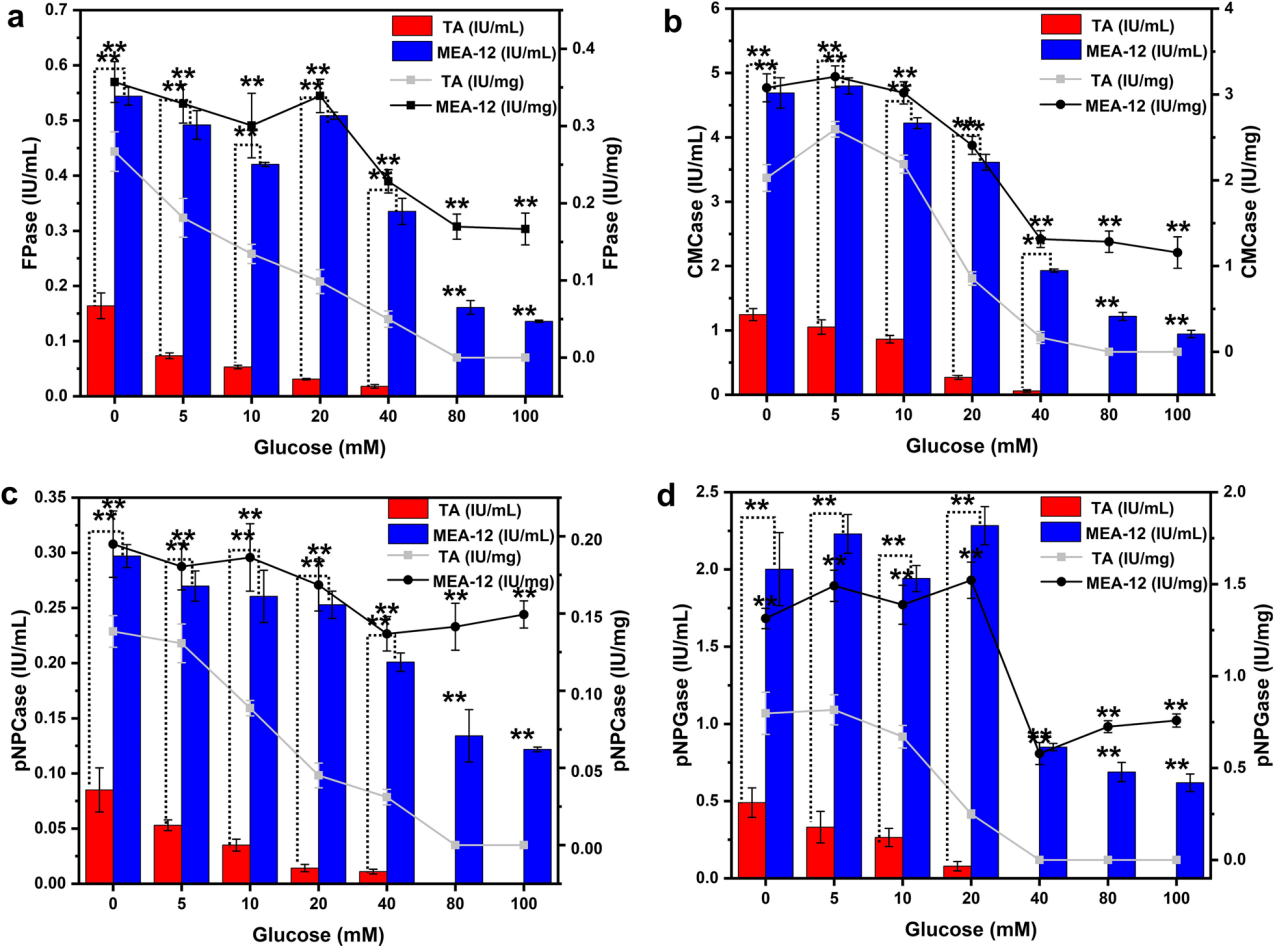
Cellulase activities of TA and MEA-12 using inducing medium supplemented with different content of glucose. “IU/mL” means the specific activities which are calculated based on volume while the “IU/mg” is based on protein mass. Error bars indicate the mean ± SD of three biological replicates and statistical significance was calculated by Student’s *t* test (* (*p* < 0.05) and ** (*p* < 0.01))

Carbon catabolite repression (CCR) is widely present in microorganism growth process [23]. When the medium contains simple carbon sources such as glucose, microorganisms preferentially use simple carbon sources and their metabolites will inhibit other non-fast-acting carbon source metabolism related gene expression and protein activity [24, 25]. Also, the inhibition of cellulase production by glucose is a common characteristic in *Trichoderma* [26]. However, there is no report on whether CCR exists in *T. afroharzianum*, as far as we know. In this study, activities of all four enzymes from parental strain TA were profoundly inhibited with the increase of glucose concentration (Fig. 4), which meant that the CCR was also present in *T. afroharzianum*. On the other hand, no significant inhibition of FPase, CMCase, pNPCase and pNPGase from mutant MEA-12 occurred by glucose reached up to 20 mM (Fig. 4). It was due to the increase in concentration of metabolic repressor in screening medium (SM1-3) during mutagenesis, allowing the mutant to gradually adapt to the high-concentration glucose culture environment. At the same time, it explained the reason for high production of mutant MEA-12 on an apparent level (glucose tolerance) and further illustrated the effectiveness of the ALE with high sugar stress.

### Total extracellular proteins and transcription levels of major cellulase structural genes and transcription factors

**Fig. 5:**
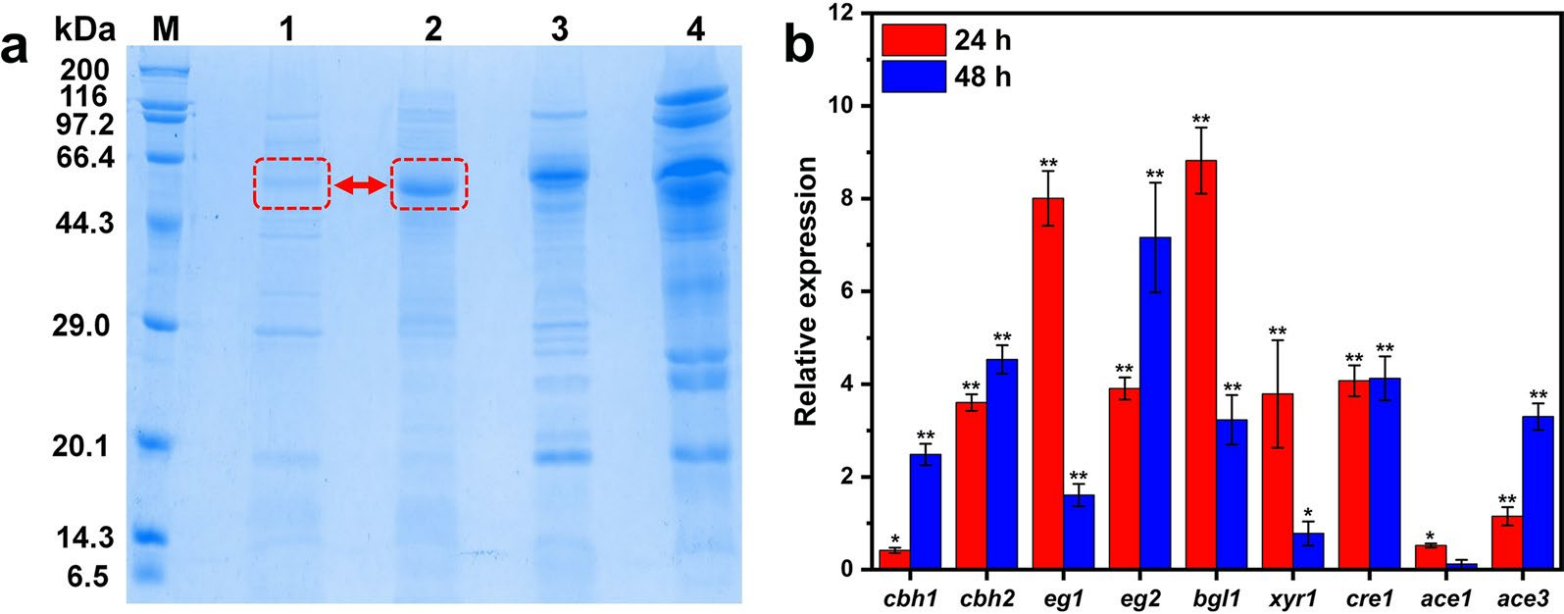
SDS-PAGE of enzyme produced by TA, MEA-12, *T. reesei* Rut-C30, Novozymes **a** and expression levels of the genes encoding major cellulase and transcription regulators in MEA-12 (**b**). Lane 1: TA; Lane 2: MEA-12; Lane 3: *T. reesei* Rut-C30; Lane 4: Novozymes. The actin gene was used as the endogenous control. Error bars indicate the mean ± SD of three biological replicates and statistical significance was calculated by Student’s *t* test (* (*p* < 0.05) and ** (*p* < 0.01))

To further analyze the differences in protein composition in different enzymes, the proteins secreted by TA, MEA-12, *T. reesei* Rut-C30 (TR) and Novozymes were compared by SDS-PAGE analysis. The composition of two protein mixtures in parent strain TA and mutant MEA-12 was similar, but the relative concentrations of different components were significantly different. Specifically, the band corresponding to cellulose-degrading enzyme cellobiohydrolase II (CBH II) (~ 60 kDa, red arrow) [27] in MEA-12 was denser than TA (Fig. 5a). Therefore, MEA-12 apparently produced more CBH II than TA. CBH II can release glucose from the non-reducing end of cellulose, and it works in concert with β-glucosidase to generate a new non-reducing end in cellulose chain, thereby improving the efficiency of hydrolysis [3]. In addition, the crude enzyme secreted by MEA-12 had achieved a good balance between different synergistic cellulase enzymes, which can degrade cellulose effectively. Furthermore, the enzyme band of *T. reesei* Rut-C30 was slightly thicker than that of MEA-12. This matched the superior power of the cellulase production model strain *T. reesei* Rut-C30. *T. reesei* Rut-C30 has been developed for decades and is currently an industrial strain with a relatively complete cellulase system [28]. The comparable protein secretion level between MEA-12 and *T. reesei* Rut-C30 and the higher production of β-glucosidase by MEA-12 (Table 2) suggest its great potential for biotechnological application, especially for lignocellulose biomass hydrolysis.

**Table 2:** Comparison of β-glucosidase activity of *T. afroharzianum* mutant MEA-12 with other microbial strains in the literature

Microorganisms	Cellulose substrate	β-glucosidase activity (IU/mL)	References
*T. reesei* QM6a	Microcrystalline cellulose and wheat bran	0.15	[33]
*T. reesei* QM9414	Microcrystalline cellulose and wheat bran	0.51	[33]
*T. *reesei Rut-C30	Microcrystalline cellulose	0.42	[34]
*T. orientalis *EU7-22	*Miscanthus* cellulose	0.49	[35]
*T. harzianum*	Carboxymethyl cellulose	0.26	[36]
*T. harzianum* LZ117	Microcrystalline cellulose and wheat bran	2.78	[33]
*T. afroharzianum*	Microcrystalline cellulose	0.52	This study
*T. afroharzianum* MEA-12	Microcrystalline cellulose	2.17	This study

In addition, transcriptional levels of major cellulase genes (*cbh1*, *cbh2*, *eg1*, *eg2* and *bgl1*) and transcriptional regulatory genes (*xyr1*, *cre1*,* ace1* and *ace3*) were measured by RT-qPCR analysis. Compared to the parental strain TA, the transcriptional levels of the major cellulase genes (*cbh1*, *cbh2*, *eg1*, *eg2*, and *bgl1*) in MEA-12 were all significantly up-regulated in both 24 h and 48 h induction and the relative expression reached 2.48, 4.54, 1.61, 7.16 and 3.23 at 48 h, respectively (Fig. 5b). Both Xyr1 and Ace3 control the inducible expression of cellulase genes in *T. harzianum* and *T. reesei*, respectively [29, 30]. Furthermore, *T. afroharzianum* is closely related to the above two strains [31]. After mutagenesis of the parental strain TA, the up-regulated transcription levels of *xyr1 *and *ace3* in MEA-12 (Fig. 5b) can partly explain its increased cellulase production. The transcription level of *cre1* at 24 h/48 h in MEA-12 was higher than that of TA, which was due to the high-yield cellulase can release more glucose from the carbon source in a short time and results in carbon metabolic repression (Fig. 5b). Ace1 is a transcription repressor that regulates cellulase production in *Trichoderma* spp., so the lower level of *ace1* transcription can improve the cellulase production [32]. However, the transcription level of *ace1* in MEA-12 was higher than that of TA after the 24 h/48 h induction. Fortunately, this increase was not obvious (24 h: 0.01 < *p* < 0.05/48 h: *p* > 0.05).

Since the whole genome analysis of *T. afroharzianum* has not been completed (https://www.ncbi.nlm.nih.gov/bioproject/PRJNA776045/), in the future, comparative genomics can be used to analyze the mechanism of mutants’ high cellulase production. Further improve cellulase production of the target strain through the inverse metabolic engineering.

### Hydrolytic analysis of different pretreated biomasses with crude enzyme produced by mutant MEA-12

Cornstalk, bamboo and reed as non-wood and non-food biomass materials, depending on their different fiber processing technology, can turn them into glucose and other wide range of products, which has been widely used in China, the United States and some of European countries [37]. Currently, *T. reesei* Rut-C30 (TR) is recognized as an excellent cellulase-producing strain. However, its lower β-glucosidase activity causes cellobiose to accumulate, which affects the efficiency of enzymatic hydrolysis and saccharification, thereby reducing the yield of ethanol [28]. Therefore, the above three kinds of biomass were chosen to evaluate the potential of cellulase produced by MEA-12, with TR as control. Furthermore, causing the high β-glucosidase (pNPGase) activity of MEA-12 (Table 2), the mixed cellulase of MEA-12 and TR was used to hydrolyze lignocellulose, which can compensate for the defects of cellulase produced by TR.

**Fig. 6:**
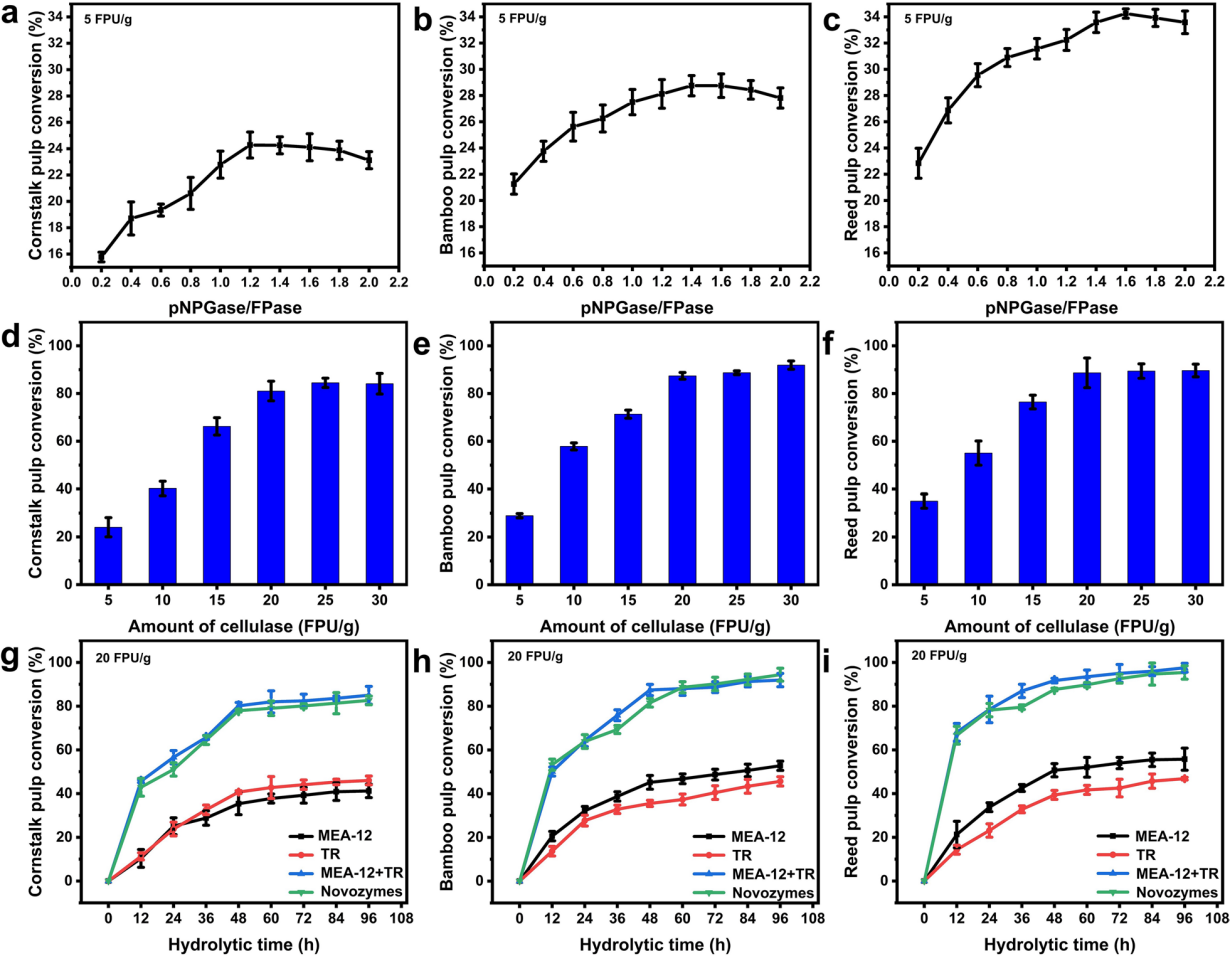
Hydrolysis efficiencies of cornstalk, bamboo and reed by crude enzymes from MEA-12, *T. reesei* Rut-C30 (TR) and Novozymes. Optimization of pNPGase/FPase in compound enzyme to hydrolyze cornstalk (**a**), bamboo (**b**) and reed (**c**). Optimization of the added amount of compound enzyme to hydrolyze cornstalk (**d**), bamboo (**e**) and reed (**f**). Hydrolysis efficiencies of the crude enzyme from MEA-12, the crude enzyme from TR, the commercial enzyme from Novozymes and the compound enzyme on cornstalk (**g**), bamboo (**h**) and reed (**i**). Error bars indicate the mean ± SD of three biological replicates

As shown in Fig. 6a–c, when cornstalk pulp was used as substrate, the enzymatic hydrolysis efficiency of compound cellulase was the highest when pNPGase/FPase (“IU/mL”: “IU/mL”)=1.2, while bamboo pulp was 1.4 and reed pulp was 1.6. This phenomenon may be attributed to the level of non-cellulose content in the substrate (cornstalk<bamboo<reed). The higher non-cellulose content hindered the process of enzymatic saccharification. Therefore, more β-glucosidase was needed to accelerate enzymatic hydrolysis and convert disaccharides into monosaccharides. When enzyme load reached 20 FPU/g, the efficiency of enzymatic hydrolysis was 81.1% and then rose slowly, which also occurred in bamboo and reed. It was because the enzymatic effect will be saturated when concentration of the enzyme was much higher than the substrate [38]. Therefore, for economic consideration, 20 FPU/g will be used as the final added amount of compound cellulase in processing substrates. Analysis of enzymatic hydrolysis reactions revealed that the hydrolytic efficiency of the crude MEA-12 enzyme for converting cornstalk pulp into glucose was slightly lower than the TR enzyme (degree of hydrolysis: 35.4% and 40.7% after 48 h, respectively Fig. 6g). In contrast, with two other biomasses as the substrate, the degree of hydrolysis was 45.2%( bamboo)/50.7% (reed) and 35.6% (bamboo)/39.4% (reed) after 48 h, with the MEA-12 and TR enzymes, respectively (Fig. 6h, i). Additionally, with cornstalk pulp as the substrate, the enzymatic hydrolysis efficiency of compound cellulase reached 80.2% after 48 h, which was slightly higher than commercial cellulase (77.9%), and it was also 1.97–2.27 times than that of single enzyme treatment (Fig. 6g). The compound cellulase exhibited strong biomass hydrolysis ability, which was also present in bamboo and reed. Finally, when hydrolyzing cornstalk, the hydrolysis efficiency of compound cellulase was up to 85.0% after 96 h (91.9% in bamboo and 97.6% in reed). It was close to or even higher than the treatment effect of commercial cellulase (82.6% in cornstalk, 94.1% in bamboo and 95.4% in reed) (Fig. 6g–i).

The enzymatic hydrolysis efficiency of pretreated biomass hinges on the composition of the enzyme mixture [39]. After mutagenesis of wild-type TA, the protein composition of cellulase changed (For example, increasing the ratio of CBH II, mentioned before) (Fig. 5a), which changed the diversity of enzymatic cocktails. Therefore, the biomass hydrolysis efficiency of the crude enzyme produced by the mutant MEA-12 had been greatly improved. Even efficiency during hydrolysis of bamboo and reed pulp exceeded the model strain TR, showing the mutant MEA-12 has high great potential for industrial application. At present, the commercial cellulase solution is mainly composed of cellulase derived from *Trichoderma* and *Aspergillus* [40]. However, the composition of cellulase from different genera is different. MEA-12, which is also of the genus *Trichoderma*, is possible to co-culture with *T. reesei* and provides a better choice for the compounding of commercial cellulase.

## Conclusion

In this study, a *T. afroharzianum* mutant strain, MEA-12, was isolated and resulted in 4.33, 6.37, 4.92 and 4.15 times increase in FPase, CMCase, pNPCase and pNPGase, respectively, with higher insensitivity for catabolite repression. Crude enzyme from *T. afroharzianum* MEA-12 showed high enzymatic hydrolysis efficiency against common biomasses (cornstalk, bamboo, reed), when combined with cellulase from *T. reesei* Rut-C30. Furthermore, the factors that improved cellulase production by MEA-12 were clarified. This study confirmed that compound mutagenesis combined with ALE method was an effective tool to increase the cellulase production of filamentous fungi and provided a new choice for processing of bioresources in the future.

## Methods

### Microorganisms and culture conditions

The *T. afroharzianum* (CICC 40189) and *T. reesei* Rut-C30 (CICC 13052) were obtained from the China Center of Industrial Culture Collection (CICC). Fresh spores were collected from *T. afroharzianum* and *T. reesei* Rut-C30 cultured on potato dextrose agar (PDA) plates for 4 days and 6 days, respectively.

For mutagenesis, *T. afroharzianum *(1×10^7^–1×10^8^ spores) were diluted and spread on the screening medium (SM), cultured at 30 ℃ for 4 days. The SM contains 2.0 g/L KH_2_PO_4_, 1.4 g/L (NH_4_)_2_SO_4_, 0.75 g/L peptone, 0.3 g/L urea, 0.3 g/L CaCl_2_, 0.3 g/L MgSO_4_·7H_2_O, 0.25 g/L yeast extract, 2.0 g/L Triton X-100, 20.0 g/L agar and 1.0 mL/L trace element nutrient solution [41]. The trace element nutrient solution contains 5.0 g/L FeSO_4_·7H_2_O, 2.0 g/L CoCl_2_·6H_2_O, 1.6 g/L MnSO_4_·4H_2_O, 1.4 g/L ZnSO_4_·7H_2_O [42]. The SM was supplemented with 10.0 g/L Carboxymethyl Cellulose-Na (CMC-Na) and 5.0 g/L 2-deoxyglucose (SM1); 3.0 g/L esculin, 0.5 g/L ferric citrate and 10.0 g/L 2-deoxyglucose (SM2); 10.0 g/L Cellulose microcrystalline (MCC) and 50.0 g/L 2-deoxyglucose (SM3).

For fermentation of enzyme production, *T. afroharzianum* (1×10^8^ spores) and *T. reesei* Rut-C30 (1×10^8^ spores) were cultured in preculture medium (20.0 g/L yeast extract, 15.0 g/L glucose, 6.0 g/L KH_2_PO_4_, 2.5 g/L (NH_4_)_2_SO_4_, 1.0 g/L CaCl_2_, 0.8 g/L MgSO_4_·7H_2_O, 2.0 g/L tween 80, 1.0 mL/L trace element nutrient solution, pH 4.8), at 180 rpm and 30 °C, for 2 days, respectively. Then, it was inoculated into fermentation medium (10.0 g/L lactose, 10.0 g/L MCC, 12.0 g/L Spray Drying Corn Steep Liquor, 1.5 g/L (NH_4_)_2_SO_4_, 1.4 g/L CaCl_2_, 1.2 g/L MgSO_4_·7H_2_O, 2.0 g/L tween 80, 1.0 mL/L trace element solution, pH 4.8) in 10% (v/v) inoculum size at 180 rpm and 30 °C, for 4 days. Centrifuge at 10,000×*g* for 15 min and collect the supernatant for measuring enzyme activity and enzymatic hydrolysis of bamboo pulp.

For RT-qPCR analyses, *T. afroharzianum* (1×10^8^ spores) were cultured in preculture medium for 2 days, then transferred into fermentation medium, at 180 rpm and 30 °C, for 24 and 48 h. Centrifuge at 12,000×*g* for 10 min to collect the mycelia and grind with liquid nitrogen to extract total RNA.

All the above media were sterilized at 121 °C for 20 min before use.

### Mutagenesis

MNNG (0.1 g) was dissolved in 1 mL acetone to prepare 10% solution for treatment. The spore suspension (970 μL) and 10% MNNG solution (30 μL) placed in a constant temperature shaker, shaking at 30 °C and 180 rpm, for 0–90 min, to determine the optimum treatment time. After the treatment of mutagenesis, the supernatant was discarded after the centrifugation at 12,000×*g* for 3 min, and the new spore suspension was prepared by washing and the precipitation with 1% normal saline for 2 times. After the dilution treatment, post-mutagenesis spore suspension was spread on the SM1 and cultured at 30 °C for 4 days. Strain with apparent positive mutation, which the ratio of transparent circle diameter to colony diameter was higher than that of the parental strain.

For EMS treatment, 50% EMS solution was prepared by mixing EMS and acetone at the volume ratio of 1:1. The spore suspension (970 μL) and 50% EMS solution (30 μL) were mixed in a constant temperature shaker, shaking at 30 °C and 180 rpm, for 0–90 min, to determine the optimum treatment time. After the treatment of mutagenesis, the supernatant was discarded by centrifugation at 12,000×*g* for 3 min, and the new spore suspension was prepared by washing and precipitation with 1% normal saline for 2 times. After the dilution treatment, post-mutagenesis spore suspension was spread on the SM2 and cultured at 30 °C for 4 days. Colony that became darker and larger in diameter was selected as the apparent positive mutation strain.

For ARTP treatment, the spore suspension (10 μL) was smeared on the slides and placed in an ARTP-IIS Bio-breeding Machine (Wuxi Tmaxtree Biotechnology Co., Ltd., China), for 0~270 s, to determine the optimum treatment time. After the mutagenesis, the slides were taken out and placed in an EP tube with 1 mL 1% normal saline. After the dilution treatment, post-mutagenesis spore suspension was spread on the SM3 and cultured at 30 °C for 4 days. Strain with apparent positive mutation was chosen according to the ratio of transparent circle diameter to colony diameter, which was higher than that of the parental strain.

After determining the optimal treatment time (50 min by MNNG, 70 min by EMS and 240 s by ARTP), perform the combined treatment as described above.

### Catabolite repression studies

For catabolite repression studies, add different concentrations of glucose (0–100 mM) to the fermentation medium. Fermentation lasted for 1–7 days and the optimum fermentation time with the highest enzyme activity was recorded for comparison.

### Enzyme activity assay

The supernatant was collected from fermented broth by centrifugation at 10,000×*g* for 15 min. Total soluble protein was quantified using the Modified Bradford Protein Assay Kit (Sangon Biotech, China) according to the manufacturer’s instructions. FPase and CMCase activities were assayed using the standard method recommended by the International Union of Pure and Applied Chemistry (IUPAC) [43]. pNPCase and pNPGase activities were assayed in a reaction system containing 0.05 M citrate buffer (pH 4.8) and 50 mM pNPC/pNPG (Sigma, USA) and then incubated at 50 °C for 30 min, respectively [44]. One unit of enzyme activity was defined as the amount of enzyme capable of releasing 1 μmol of reducing sugar or pNP per min under the conditions of 50 °C and pH 4.8. Furthermore, the specific activities (IU/mg) = Enzyme activity (IU/mL) / Total soluble protein (mg/mL). The FPase, CMCase, pNPCase and pNPGase activities represent the total cellulase, endoglucanase, cellobiohydrolase and β-glucosidase activity, respectively [45].

### SDS-PAGE and RT-qPCR

Proteins secreted by *T. afroharzianum* and MEA-12 cultured in fermentation medium were collected at the 4th day and analyzed by sodium dodecyl sulfate-polyacrylamide gel electrophoresis (SDS-PAGE).

Culture conditions of the strains used for RT-qPCR were same to those described above. Total RNA was extracted using the RNA pure total RNA rapid extraction kit (Shanghai Hlingene Biological technology Co., Ltd., China) and then cDNA synthesis using the *TransScript*® One-Step gDNA Removal and cDNA Synthesis SuperMix (TransGen Biotech, China) according to the manufacturer's instructions. RT-qPCR primers of the tested genes were designed as described in a previous study (Additional file 1: Table S1), and it was performed using the ChamQ™ Univerasl SYBR qPCR Master Mix (Vazyme, China) and the QuantStudio™ real-time PCR system (Thermo Fisher Scientific, USA) according to the manufacturer's instructions. Relative quantification of the tested genes was calculated by using comparative cycle threshold (2^−ΔΔCT^) method. The actin gene was chosen as the endogenous control to normalize the expression levels. All experiments were performed in triplicates.

### Enzymatic hydrolysis of pretreated biomass

Cornstalk pulp (contains 72.7% cellulose, 20.1% hemicellulose, 7.2% others), bamboo pulp (contains 71.9% cellulose, 21.4% hemicellulose, 6.7% others) and reed pulp (contains 66.6% cellulose, 27.3% hemicellulose, 6.1% others) (Additional file 1: Fig. S4) produced by cooking with active oxygen and solid alkali (CAOSA), a novel pretreatment method reported by the authors’ group [46]. Raw material (1 kg, dry weight), MgO (150 g) and water (4 kg) were mixed in a spherical digester (designed by authors’ group and built by Yantai Keli Chemical Equipment Co., Ltd., China) at an initial oxygen pressure of 2.0 MPa and a temperature of 160 °C for about 10 h. After the reaction, the solid pulp was filtered and washed with clean water several times. The obtained pulp was used as the substrate for enzymatic saccharification. Under inducing conditions, fermentation was carried out to produce crude enzyme, which was subsequently used for the enzymatic hydrolysis of different biomasses and compared with commercial enzymes (Cellic® CTec2 (Novozymes A/S, Damark)). Enzymatic hydrolysis was performed in 0.05 M citrate buffer (pH 4.8) at 2.0% (g: mL) substrate consistency and then incubated at 50 °C and 180 rpm for 96 h [47]. The enzyme loading was 5 FPU/g substrate (dry weight), which would be optimized later. The hydrolysates were sampled every 12 h, boiled for 5 min to inactivate the enzyme, and then centrifuged at 10,000×g. Glucose in the supernatant was determined using an SBA-40E Bio-sensor (Jinan Yanhe Biotechnology Co., Ltd., China). Biomass conversion (%) = (Glucose (g/L) × Enzymatic hydrolysis system (*L*) × 0.9)/(Substrate (*g*)×Cellulose content (%)).

### Statistical analysis

All the experiments values presented in figures are mean±SD calculated with Excel 2019. All experiments were conducted in triplicate and all the multiple comparison tests were performed with Student’s *t* test (significance levels: * (*p*<0.05) and ** (*p*<0.01)).

## Supplementary Information

**Additional file 1: Table S1.** Primers used for RT-qPCR. **Figure S1.** Enzyme activity of MNNG mutagenesis re-screening. **a** diameter ratio (hydrolysis circle diameter/strain diameter) of TA and its mutants, **b** FPase activities of TA and its mutants, **c** CMCase activities of TA and its mutants. **Figure S2.** Enzyme activity of EMS mutagenesis re-screening. **a** FPase activities of M-84 and its mutants, **b** CMCase activities of M-84 and its mutants, **c** pNPCase activities of M-84 and its mutants, **d** pNPGase activities of M-84 and its mutants. **Figure S3.** Enzyme activity of ARTP mutagenesis re-screening. **a** FPase activities of ME-10 and its mutants, **b** CMCase activities of ME-10 and its mutants, **c** pNPCase activities of ME-10 and its mutants, **d** pNPGase activities of ME-10 and its mutants. **Figure S4.** Common biomasses after pretreatment for hydrolysis.

## Data Availability

All data and materials are available in the main text and supporting information.

## Abbreviations

CCR: Carbon catabolite repression
MNNG: N-methyl-N’-nitro-N-nitrosoguanidine
EMS: Ethyl Methanesulfonate
ARTP: Atmospheric and Room Temperature Plasma
ALE: Adaptive laboratory evolution
CAOSA: Cooking with active oxygen and solid alkali
SM: Screening medium
SDS-PAGE: Sodium dodecyl sulfate-polyacrylamide gel electrophoresis
RT-qPCR: Real-time reverse transcription quantitative PCR
EGs: Endoglucanases
CBHs: Cellobiohydrolases
BGs: β-Glucosidases
TA: *T. afroharzianum*
TR: *T. reesei* Rut-C30
