# Supplementary material for: Cellulase production and efficient saccharification of biomass by a new mutant Trichoderma afroharzianum MEA-12
Source: Biotechnol Biofuels. 2021 Nov 22;14:219. doi: 10.1186/s13068-021-02072-z (PMC8607671; doi:10.1186/s13068-021-02072-z)
Supplement: Supplementary file 1 — Additional file 1: Table S1. Primers used for RT-qPCR. Figure S1. Enzyme activity of MNNG mutagenesis re-screening. a diameter ratio (hydrolysis circle diameter/strain diameter) of TA and its mutants, b FPase activities of TA and its mutants, c CMCase activities of TA and its mutants. Figure S2. Enzyme activity of EMS mutagenesis re-screening. a FPase activities of M-84 and its mutants, b CMCase activities of M-84 and its mutants, c pNPCase activities of M-84 and its mutants, d pNPGase activities of M-84 and its mutants. Figure S3. Enzyme activity of ARTP mutagenesis re-screening. a FPase activities of ME-10 and its mutants, b CMCase activities of ME-10 and its mutants, c pNPCase activities of ME-10 and its mutants, d pNPGase activities of ME-10 and its mutants. Figure S4. Common biomasses after pretreatment for hydrolysis. [file 13068_2021_2072_MOESM1_ESM.docx]

**Additional file 1**

**Cellulase Production and Efficient Saccharification of Biomass by a New Mutant *Trichoderma afroharzianum* MEA-12**

Zhi-Qing Peng^a^, Chuang Li^a^, Yi Lin^a^, Sheng-Shan Wu^a,b,c^, Li-Hui Gan^a,b,c^, Jian Liu^a,b,c^, Shu-Liang Yang^a,b,c^, Xian-Hai Zeng^a,b,c*^, Lu Lin^a,b,c^

^a^College of Energy, Xiamen University, Xiamen 361102, China

^b^Fujian Engineering and Research Centre of Clean and High-valued Technologies for Biomass, Xiamen 361102, China

^c^Xiamen Key Laboratory of Clean and High-valued Utilization for Biomass, Xiamen 361102, China

*Corresponding author: xianhai.zeng@xmu.edu.cn (Xian-Hai Zeng)

Table S1. Primers used for RT-qPCR.

| **Primers** | | **Sequences (5’-3’)** |
| --- | --- | --- |
| *cbh1* | Fwd | ATCGGCTTCGTCACGCAATC |
|  | Rvs | ACGCCACCATCCGCATCCA |
| *cbh2* | Fwd | GACAAACCTCGGCACTCC |
|  | Rvs | GACCAGCGTCCAGATACATT |
| *eg1* | Fwd | CAGGGCTTCTGCTGTAATGAG |
|  | Rvs | TTGAACTGGGTGATGATGGTG |
| *eg2* | Fwd | GCTCCGCCAGAATAACCG |
|  | Rvs | CAGCCAACATAGCCAAGATAGAC |
| *bgl1* | Fwd | ATCACCTACCCGCCTTCA |
|  | Rvs | TCTCGTCGTCGGATGTTG |
| *xyr1* | Fwd | TGCTTGACGACGACGACTTGT |
|  | Rvs | ACGCCGTAGAAGAAGGGCAT |
| *cre1* | Fwd | TCTACGGCTCCTTCTTCTC |
|  | Rvs | ACAAGTTCCTCAGACTCGG |
| *ace1* | Fwd | TGATGAGGGCTTTGACGAGTC |
|  | Rvs | GGTTGAAGATGTCGGGCTGT |
| *ace3* | Fwd | TGCTGAGGGTGATGAACGAG |
|  | Rvs | GGGTGAATCCTGGTTGCGAT |
| *actin* | Fwd | TCCATCATGAAGTGCGAC |
|  | Rvs | GTAGAAGGAGCAAGAGCAGTG |


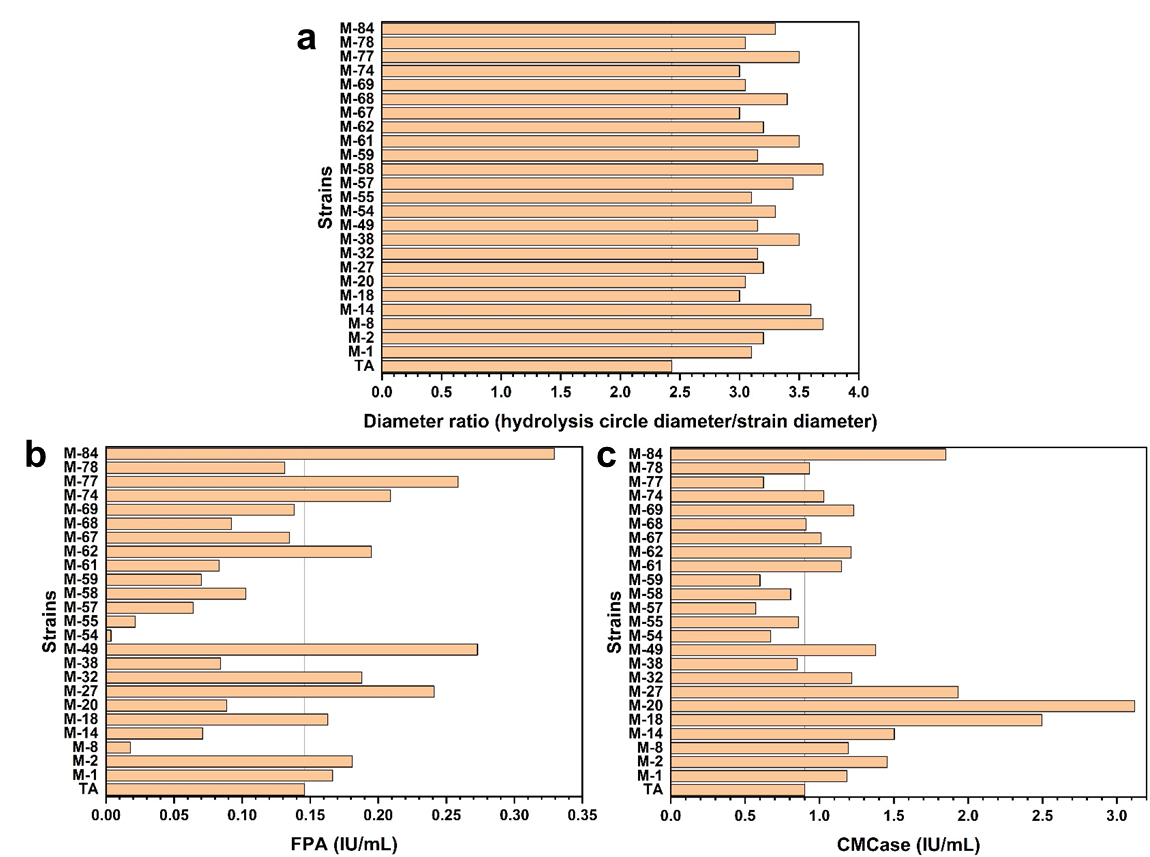


**Figure S1.** Enzyme activity of MNNG mutagenesis re-screening. (a): diameter ratio (hydrolysis circle diameter/strain diameter) of TA and its mutants, (b): FPase activities of TA and its mutants, (c): CMCase activities of TA and its mutants.


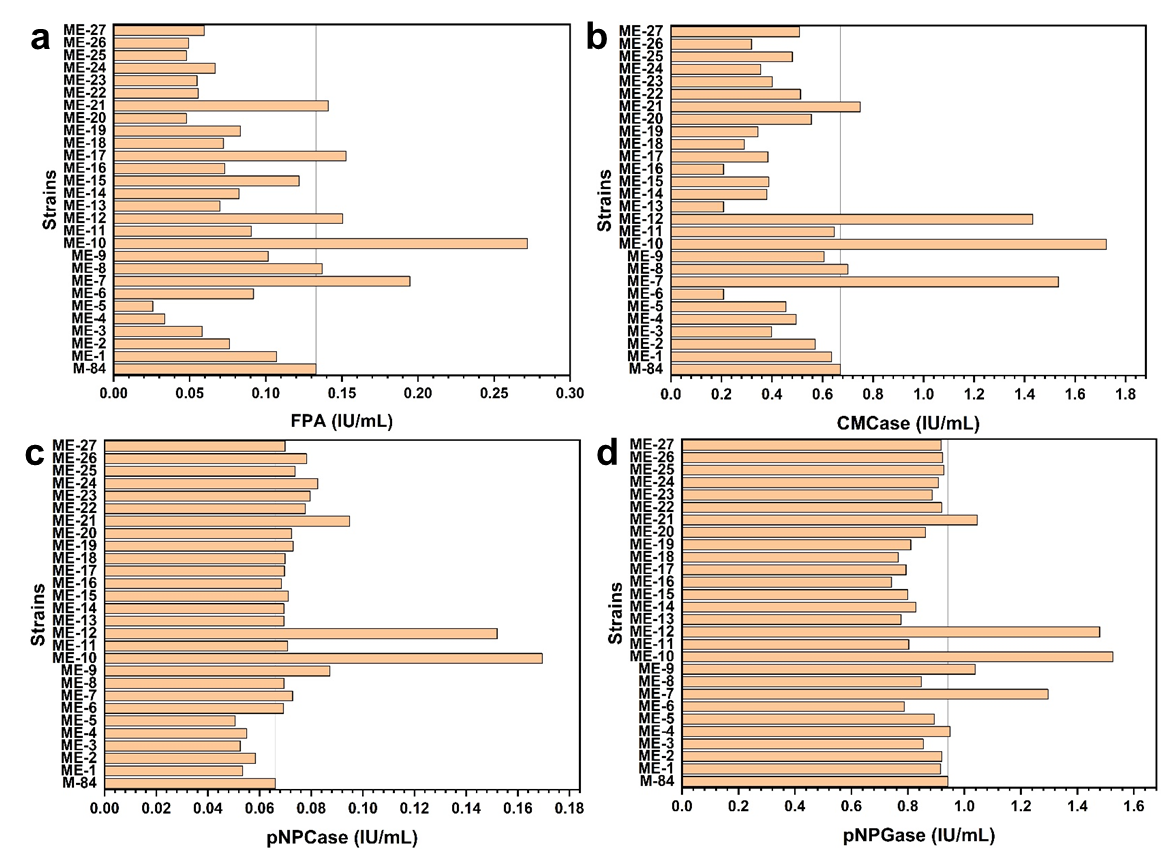


**Figure S2.** Enzyme activity of EMS mutagenesis re-screening. (a): FPase activities of M-84 and its mutants, (b): CMCase activities of M-84 and its mutants, (c): pNPCase activities of M-84 and its mutants, (d): pNPGase activities of M-84 and its mutants.


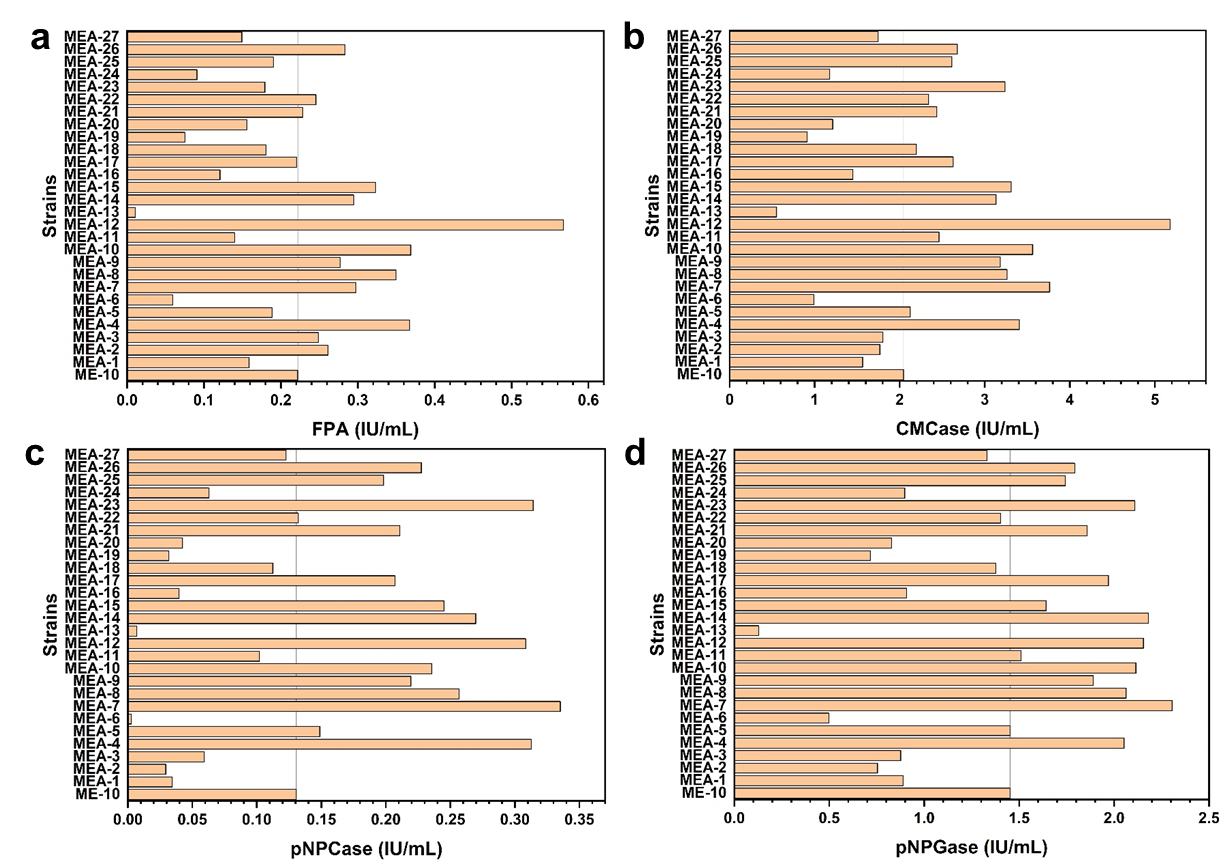


**Figure S3.** Enzyme activity of ARTP mutagenesis re-screening. (a): FPase activities of ME-10 and its mutants, (b): CMCase activities of ME-10 and its mutants, (c): pNPCase activities of ME-10 and its mutants, (d): pNPGase activities of ME-10 and its mutants.


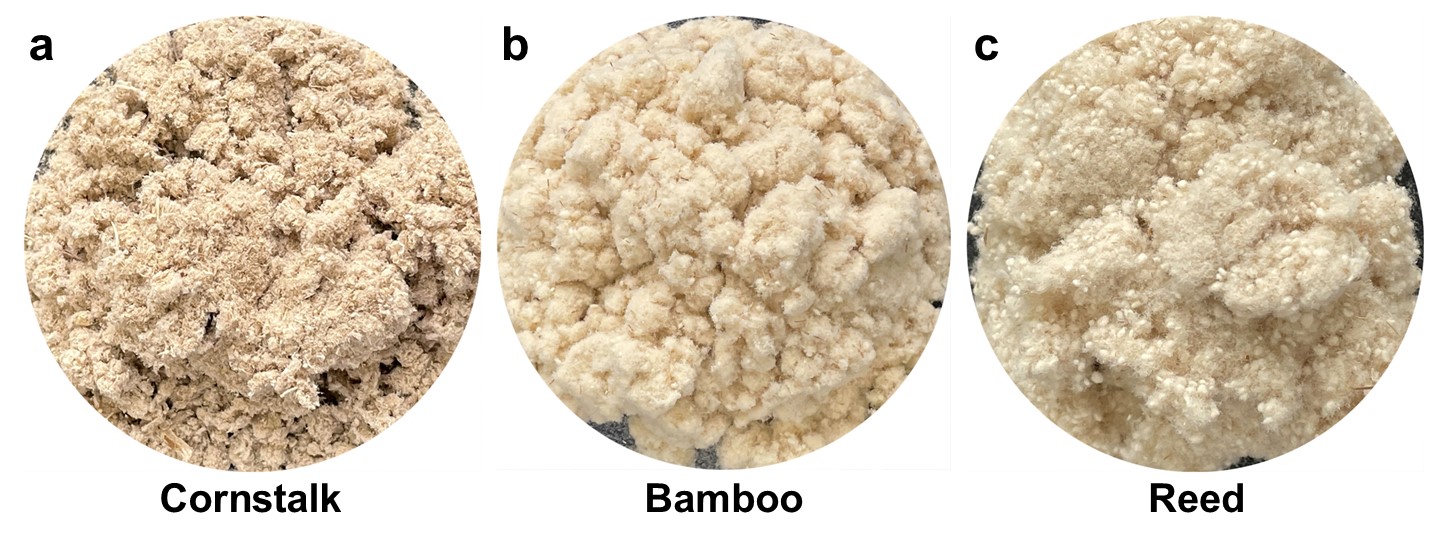


**Figure S4.** Common biomasses after pretreatment for hydrolysis.
